# Correlation between changes in apathy and cognition in Alzheimer’s disease associated apathy: analysis of the Apathy in Dementia Methylphenidate Trial 2 (ADMET 2)

**DOI:** 10.1002/alz.091793

**Published:** 2025-01-03

**Authors:** Krushnaa Sankhe, Shankar Tumati, Jamie Perin, Luc Rivet, Danielle Vieira, Paul B. Rosenberg, Nathan Herrmann, David Shade, Alan J. Lerner, Prasad R Padala, Olga Brawman‐Mintzer, Christopher H. van Dyck, Anton P. Porsteinsson, Suzanne Craft, Allan I. Levey, Jacobo Mintzer, Krista L. Lanctôt

**Affiliations:** ^1^ Sunnybrook Research Institute, University of Toronto, Toronto, Canada, Toronto, ON Canada; ^2^ Sunnybrook Research Institute, Toronto, ON Canada; ^3^ Johns Hopkins, Baltimore, MD USA; ^4^ University of Toronto, Toronto, ON Canada; ^5^ Johns Hopkins University School of Medicine, Baltimore, MD USA; ^6^ Sunnybrook Health Sciences Centre, Toronto, ON Canada; ^7^ Department of Neurology, University Hospitals Cleveland Medical Center, Cleveland, OH USA; ^8^ University of Arkansas for Medical Sciences, Little Rock, AR USA; ^9^ Central Arkansas Veterans Healthcare System, North Little Rock, AR USA; ^10^ Medical University of South Carolina, Charleston, SC USA; ^11^ Alzheimer’s Disease Research Unit, Yale School of Medicine, New Haven, CT USA; ^12^ University of Rochester School of Medicine and Dentistry, Rochester, NY USA; ^13^ Wake Forest University School of Medicine, Winston‐Salem, NC USA; ^14^ Emory University, Atlanta, GA USA

## Abstract

**Background:**

Apathy in Alzheimer’s disease (AD) is associated with cognitive impairment, particularly executive functions such as selective attention, making it unclear whether apathy should be a separate treatment target. Apathy in Dementia Methylphenidate Trial 2 (ADMET 2) is the largest and most recent trial assessing apathy and cognition. This analysis assessed whether changes in apathy correlated to changes in various cognitive domains in ADMET 2.

**Method:**

In this multicentre, randomized, placebo‐controlled trial, 200 participants with mild to moderate AD received 20 mg methylphenidate (MPH) or placebo (PLB) for 6 months. The Neuropsychiatric Inventory apathy (NPI‐A) subscale measured apathy. Cognitive tests included Mini‐Mental State Exam (MMSE), Hopkins Verbal Learning‐immediate (HVLT‐I) and delayed (HVLT‐D), Digit Span forward (DF) and backward (DB), Trail Making (TMT A and B), Action Verbal Fluency (AV), Category Fluency (CF), and Boston Naming Test (BNT). Linear mixed models were conducted with cognitive change scores as the dependent variable and time and change in NPI‐A as the independent variable, overall and in each treatment group. All models were adjusted for baseline cognitive test score and NPI‐apathy, age, sex, diabetes, and treatment group.

**Result:**

199 ADMET 2 participants (131 (66%) male) were included. For all participants, worsening apathy was associated with worsening MMSE (B (SE) = ‐0.10 (0.03), p = 0.003), CF (B (SE) = ‐0.13 (0.04), p = 0.0009), and HVLT‐I (B (SE) = ‐0.13 (0.05), p = 0.005). In the MPH group (n = 98), worsening apathy was associated only with worsening CF (B (SE) = ‐0.21 (0.06), p = 0.0008). In the PLB group (n = 101), worsening apathy was associated with worsening MMSE (B (SE) = ‐0.13 (0.05), p = 0.006), HVLT‐I (B (SE) = ‐0.30 (0.06), p = 0.000002), and HVLT‐D (B (SE) = ‐0.06 (0.02), p = 0.002). Changes in apathy were not associated with performance on the remaining cognitive tests.

**Conclusion:**

Changes in apathy are associated with changes in cognition as part of the natural history of AD. However, this association is disrupted by MPH treatment and suggests that improvements in apathy in AD with MPH were not driven by cognitive changes. These results are consistent with the view that apathy as a syndrome is related to, but distinct from cognition.

